# Specific subsystems of the inferior parietal lobule are associated with hand dysfunction following stroke: A cross‐sectional resting‐state fMRI study

**DOI:** 10.1111/cns.13946

**Published:** 2022-08-23

**Authors:** FeiWen Liu, ChangCheng Chen, ZhongFei Bai, WenJun Hong, SiZhong Wang, ChaoZheng Tang

**Affiliations:** ^1^ Department of Rehabilitation Medicine Chengdu Second People's Hospital Chengdu China; ^2^ Department of Rehabilitation Medicine Qingtian People's Hospital Lishui China; ^3^ Yangzhi Rehabilitation Hospital Affiliated to Tongji University (Shanghai Sunshine Rehabilitation Center) Shanghai China; ^4^ Department of Rehabilitation Medicine, Nanjing Drum Tower Hospital The Affiliated Hospital of Nanjing University Medical School Nanjing China; ^5^ Centre for Health, Activity and Rehabilitation Research (CHARR), School of Physiotherapy University of Otago Dunedin New Zealand; ^6^ Capacity Building and Continuing Education Center National Health Commission of the People's Republic of China Beijing China

**Keywords:** effective connectivity, Granger causality analysis, hand dysfunction, inferior parietal lobule, resting‐state functional magnetic resonance imaging, stroke

## Abstract

**Aim:**

The inferior parietal lobule (IPL) plays important roles in reaching and grasping during hand movements, but how reorganizations of IPL subsystems underlie the paretic hand remains unclear. We aimed to explore whether specific IPL subsystems were disrupted and associated with hand performance after chronic stroke.

**Methods:**

In this cross‐sectional study, we recruited 65 patients who had chronic subcortical strokes and 40 healthy controls from China. Each participant underwent the Fugl‐Meyer Assessment of Hand and Wrist and resting‐state fMRI at baseline. We mainly explored the group differences in resting‐state effective connectivity (EC) patterns for six IPL subregions in each hemisphere, and we correlated these EC patterns with paretic hand performance across the whole stroke group and stroke subgroups. Moreover, we used receiver operating characteristic curve analysis to distinguish the stroke subgroups with partially (PPH) and completely (CPH) paretic hands.

**Results:**

Stroke patients exhibited abnormal EC patterns with ipsilesional PFt and bilateral PGa, and five sensorimotor‐parietal/two parietal–temporal subsystems were positively or negatively correlated with hand performance. Compared with CPH patients, PPH patients exhibited abnormal EC patterns with the contralesional PFop. The PPH patients had one motor‐parietal subsystem, while the CPH patients had one sensorimotor‐parietal and three parietal‐occipital subsystems that were associated with hand performance. Notably, the EC strength from the contralesional PFop to the ipsilesional superior frontal gyrus could distinguish patients with PPH from patients with CPH.

**Conclusions:**

The IPL subsystems manifest specific functional reorganization and are associated with hand dysfunction following chronic stroke.

## Graphical abstract

Stroke patients with hand dysfunction suffer from poor quality of life. Considering that the inferior parietal lobule (IPL) plays important roles in reaching and grasping during hand movements, investigating how the IPL subsystems are reorganized following stroke could contribute to understanding the mechanisms underlying hand dysfunction. Here, we report that stroke patients have five sensorimotor‐parietal/two parietal‐temporal subsystems that were positively or negatively correlated with hand performance. Moreover, we also found that patients with a partially paretic hand (PPH) have one motor‐parietal subsystem, while patients with a completely paretic hand (CPH) have one sensorimotor‐parietal subsystem and three parietal‐occipital subsystems were associated with hand performance. Notably, the EC strength from the contralesional PFop to the ipsilesional superior frontal gyrus could distinguish patients with PPH from patients with CPH. These findings imply that specific reorganizations of IPL subsystems are associated with hand dysfunction after chronic stroke, which might be helpful for the development of personalized neurorehabilitation.

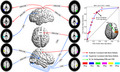

## 1 INTRODUCTION

Stroke is a primary cause of disability worldwide,^1^ and hand dysfunction severely affects the independent ability of stroke survivors.^2^ Motor dysfunction following stroke results from widespread disconnection within the sensorimotor networks^3^ and is mainly driven by interhemispheric disruptions among the bilateral central sulcus, secondary somatosensory cortex, supplementary motor area (SMA), thalamus, putamen and cerebellum,^4^ suggesting an important role of interhemispheric coordination.^5^ Connectivity analyses based on resting‐state functional magnetic resonance imaging (rs‐fMRI) have provided a useful framework for acquiring insights into the pathophysiological mechanisms of hand dysfunction after stroke.^6^, ^7^, ^8^

Converging evidence has suggested that the inferior parietal lobule (IPL) involves reaching, grasping and force adaptation during hand movements.^9^, ^10^ For example, damage to the right IPL impedes leftward hand movements toward visual targets.^11^ Our previous studies have shown that hand dysfunction is characterized by disrupted connectivity patterns with the parietal lobe.^12^, ^13^ Further subgroup analyses revealed that patients with good hand performance showed reduced causal influence from the ipsilesional IPL to the ipsilesional premotor cortex and primary somatosensory cortex.^14^ Moreover, the integrity of the frontoparietal fibers was associated with motor dysfunction,^15^ and increased spontaneous brain activity within the ipsilesional IPL was positively correlated with motor imagery‐induced functional recovery.^16^ Recently, changes in the connectivity of IPL subregions have received increasing attention due to their structural and functional heterogeneity.^17^, ^18^, ^19^ Selective disconnection of the IPL subregions has been reported in patients with schizophrenia^20^ and Alzheimer's disease.^21^ Therefore, studies exploring the altered connectivity of the IPL subregions could contribute to clarifying the neural basis of hand dysfunction after stroke.

Effective connectivity (EC) analysis has been used to detect the causal influences among brain regions beyond the functional connectivity analysis.^8^ Using dynamic causal modeling, previous studies have reported that normalized causal influences among core sensorimotor nodes are associated with motor recovery.^22^, ^23^, ^24^ When using Granger causality analysis, Gao et al. demonstrated that the left IPL is a key causal source associated with the tasks of motor execution and motor imagery.^25^ Furthermore, based on rs‐fMRI and Granger causality analyses, our recent study found that EC from the ipsilesional primary motor cortex_4a to the ipsilesional superior parietal lobe was negatively associated with hand performance in patients experiencing chronic stroke.^14^ However, to date, the extent to which IPL subregions interact with other brain regions that might underlie hand dysfunction has remained unclear in the context of chronic stroke.

To address this issue, we studied the resting‐state EC patterns between IPL subregions and the whole brain in a homogeneous sample of patients with chronic left subcortical stroke and probed their associations with paretic hand performance. Furthermore, we performed a pilot receiver operating characteristic (ROC) curve analysis to determine whether specific EC patterns could distinguish stroke patients with different hand dysfunction.

## 2 METHODS

### 2.1 Subjects

**FIGURE 1:**
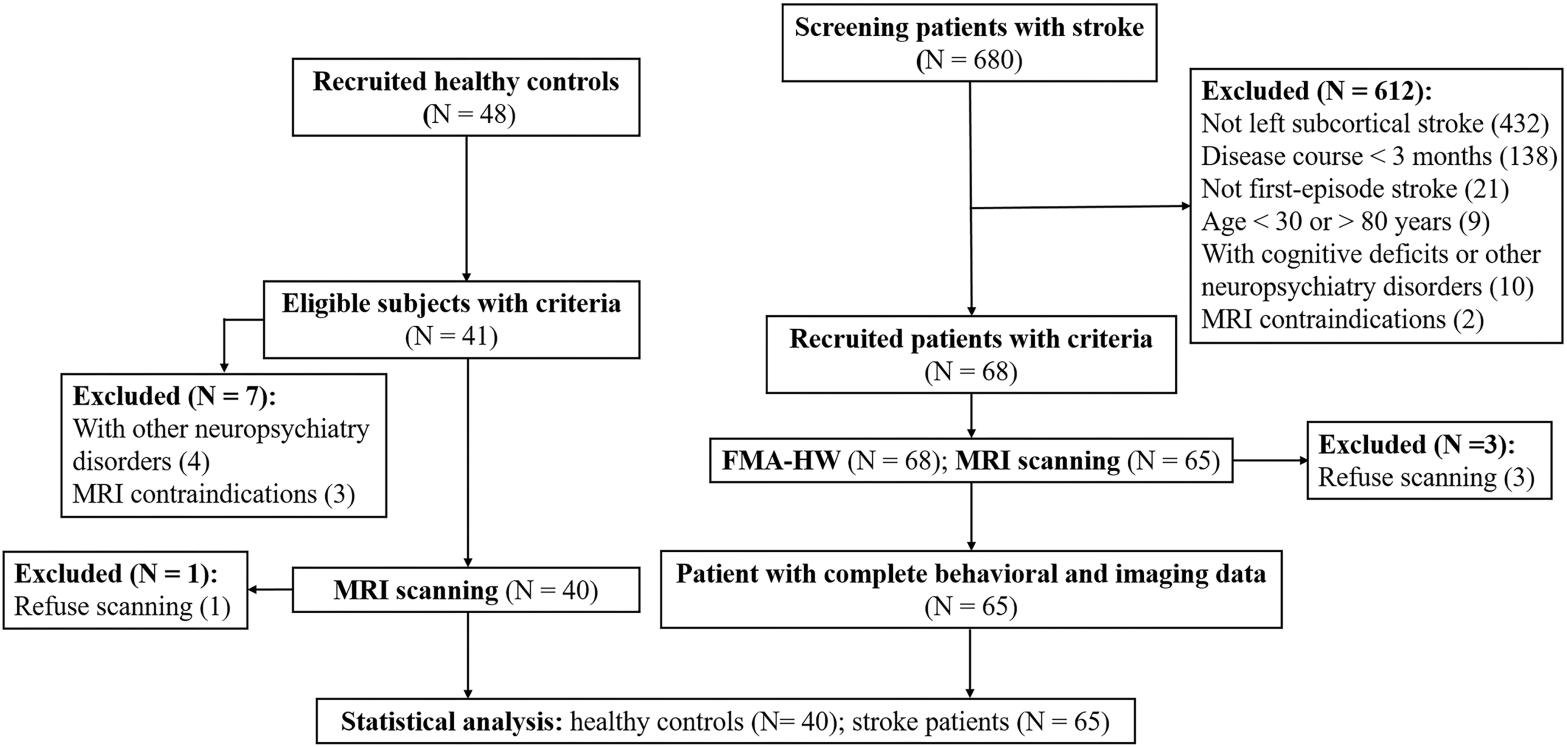
A flow chart shows the whole process of enrollment in this study

In this cross‐sectional study, patients experiencing chronic stroke restricted to the left subcortex and healthy controls were prospectively admitted from the local hospital and community from June 2010 to June 2017 in China. Each participant underwent one assessment of paretic hand performance and resting‐state fMRI (see 2.2 and 2.3 for details). This project was approved by the hospital ethics committee, and written informed consent was obtained from each participant. Detailed criteria for the inclusion and exclusion of patients and controls were described in our previous study.^14^ As shown in Figure 1, the flow chart provides detailed information about the screening, inclusion and exclusion criteria for all of the participants analyzed in this study.

To allow for the direct comparison of stroke patients with different hand dysfunction, we used the Paretic Hand Scale (see Supporting Materials) to divide stroke patients into the partially (PPH) and completely (CPH) paretic hand subgroups.^13^, ^14^ Stroke patients who were able to complete one or more tasks were classified as having PPH, while those who were unable to complete any task were classified as having CPH.

### 2.2 Assessment of paretic hand performance

The hand dysfunction of all chronic stroke patients was assessed by a masked rehabilitation physician using the modified Hand and Wrist subscale of the Fugl‐Meyer Assessment (FMA‐HW)^14^ before collecting imaging data. The FMA‐HW subscale consists of a wrist section (five items) and a hand section (seven items) with a possible score ranging from 0 to 24 points, and it was considered the primary measurement.

### 2.3 Acquisition of imaging data

All images were acquired using a 3‐T scanner (Siemens Trio, Germany). T1‐weighted images were obtained using an MPRAGE sequence: 192 sagittal slices, 1 mm slice thickness, 0.5 mm gap, 1900/3.42/900 ms TR/TE/TI, 240 × 240 FOV, 9° flip angle, and 256 × 256 matrix size. T2‐weighted images were obtained using a TSE sequence: 30 axial slices, 5 mm slice thickness, without gap, 6000/93 ms TR/TE, 220 × 220 FOV, 120° flip angle, and 320 × 320 matrix size. Functional images were obtained using an EPI sequence: 30 axial slices, 4 mm slice thickness, 0.8 mm gap, 2000/30 ms TR/TE, 220 × 220 FOV, 90° flip angle, 64 × 64 matrix size, 240 volumes, and scanning time 8 min 6 s (the first 6 s were for dummy scanning). During scanning, each participant was guided to keep the eyes closed, the mind relaxed and the head motionless.

### 2.4 Mapping the lesion frequency map

**FIGURE 2:**
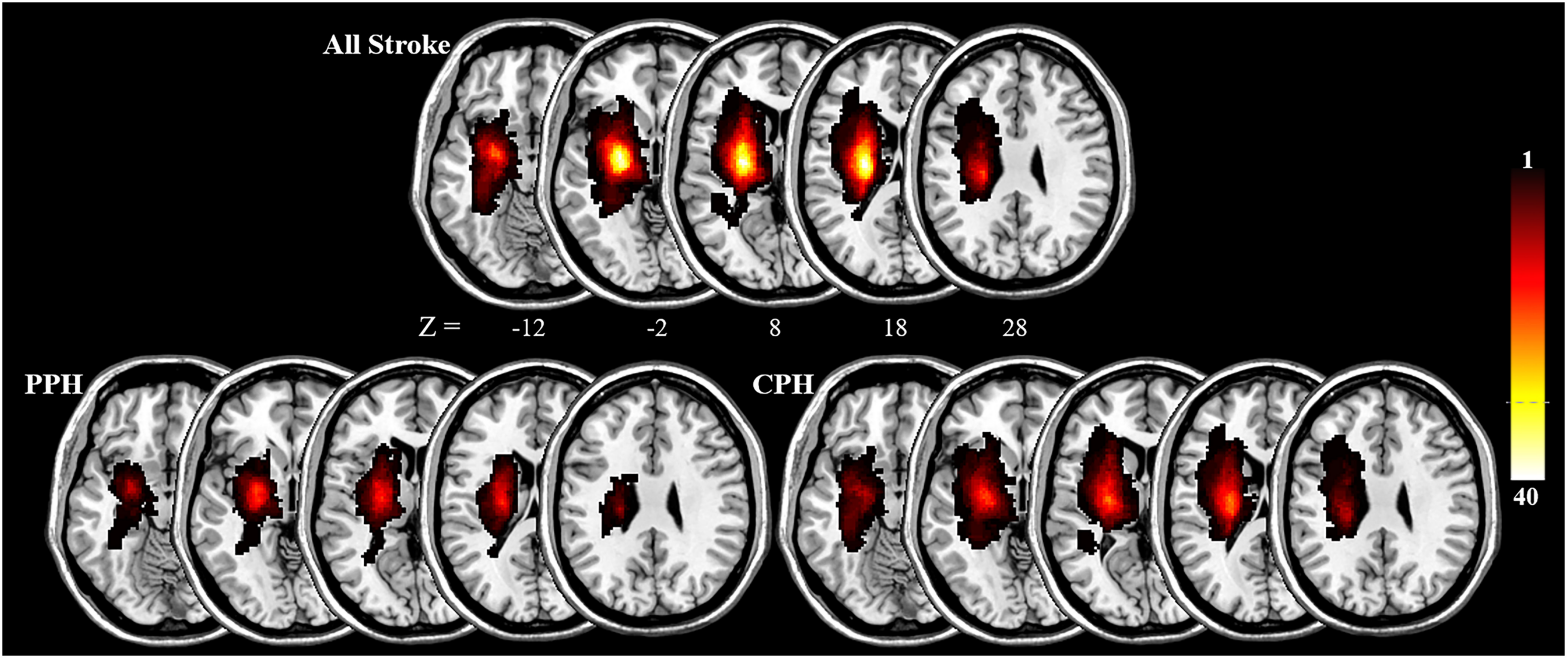
Lesion distribution map for stroke, PPH, and CPH patients. The color bar indicates the frequency of patients presenting with lesions in each voxel in the left (ipsilesional) hemisphere. CPH, completely paretic hand; PPH, partially paretic hand

We first used the MRIcron (https://people.cas.sc.edu/rorden/mricron/; version V1.0.20190902) to delineate the lesion profiles of each stroke patient on T2‐weighted images (see Supporting Materials). Then, the T2‐weighted lesion masks of all stroke patients were standardized to the Montreal Neurological Institute space. Finally, we totaled each resampled lesion mask with a resolution of 1 × 1 × 1 mm^3^ to create the lesion overlap map (Figure 2).

### 2.5 Preprocessing of neuroimaging data

We used DPABI software (http://rfmri.org/dpabi; version V5.0_201001) to preprocess the imaging data, and the steps included: (1) deleting the first 10 time points of 240 volumes; (2) correcting the slice timing; (3) realigning the head motion; (4) standardizing to the Montreal Neurological Institute space by the lesion masks^26^ and unified segmentation of structural images^27^; (5) spatially smoothing (FWHM = 6 mm); (6) linearly detrending; and (7) bandpass filtering (0.01–0.1 Hz). Finally, we regressed out the Friston 24 head motion parameters and other noise signals (e.g., derived from the global brain, cerebrospinal fluid, and white matter). During image preprocessing, no participants were excluded based on the predefined criteria of head motion (exceeding 2 mm/degree). To control for the potential influences of head motion on the results, we regressed out the framewise displacement in all subsequent statistical analyses.^28^

### 2.6 Definition of the IPL subregions and EC analysis

**FIGURE 3:**
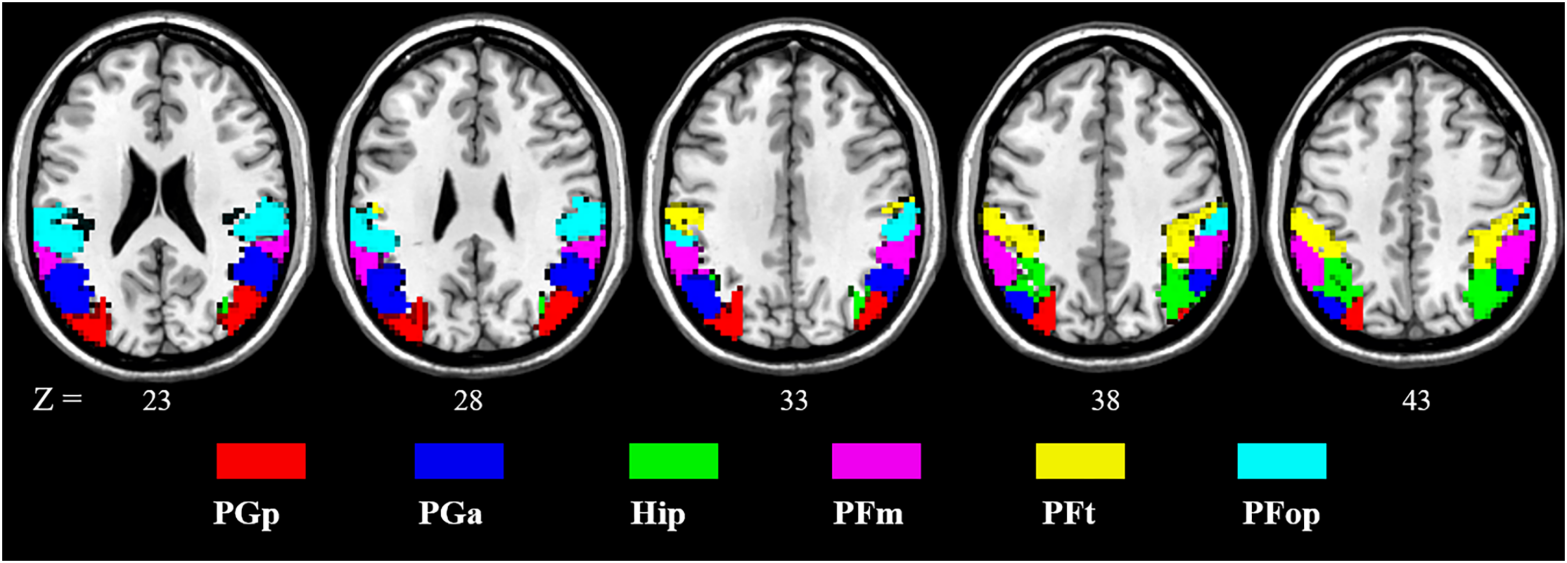
Twelve seeds were defined by subregions of the inferior parietal lobule according to the Brainnetome atlas. Each hemisphere has six subregion seeds, and the left hemisphere corresponds to the ipsilesional side

Six IPL subregions were defined in the ipsilesional and contralesional hemispheres based on the Brainnetome atlas,^29^ which is spatially similar to previous cytoarchitectonic, functional and anatomical parcellations.^18^, ^19^, ^30^, ^31^ For each hemisphere, the six IPL subregions included A39c (PGp), A39rd (Hip), A39rv (PGa), A40c (PFm), A40rd (PFt) and A40rv (PFop) (Figure 3).

We used RESTplus software (http://restfmri.net/forum/restplus; version V1.24) to calculate the seed‐based voxelwise EC maps. First, a coefficient‐based Granger causality analysis was conducted to obtain the EC maps for all participants and then were converted to z values using Fisher's r‐to‐z transformation. Next, the one‐sample *t* test was used to obtain the group EC patterns of each IPL subregion. BrainNet Viewer (www.nitrc.org/projects/bnv; version 1.7) was used to visualize the results.

### 2.7 Statistical analysis

Our primary goal was to investigate the specifically disrupted EC patterns of IPL subregions and their associations with hand dysfunction in all stroke patients and stroke subgroups, while classifying different hand dysfunctions by ROC analysis was our secondary goal. We first used SPSS software (version 25.0, IBM Inc.) to evaluate the normality of continuous variables (Shapiro–Wilk test), followed by the two independent samples *t*‐test or the Mann–Whitney test depending on whether the variables were distributed normally. For classification variables (gender and stroke type), we employed the χ^2^ test to analyze the differences between groups. The two‐sample t test was used to explore EC differences between all stroke patients and healthy controls, with gender, age, and framewise displacement as covariates. For subgroup analyses, we also regressed out the duration of illness and lesion volume. Multiple regression analysis was performed to explore associations between the EC patterns of each IPL subregion and the FMA‐HW scores in all stroke patients, with gender, age, framewise displacement, duration of illness, and lesion volume as covariates. We also performed the same analysis on both the PPH and CPH subgroups. The statistical threshold was set at *p* < 0.001 with a cluster size of 64–82 (for between‐group analysis) or 46–74 voxels (for multiple regression analysis) within a gray matter mask (cluster size varied for distinct subregions), corresponding to a corrected *p* < 0.001. For surviving brain regions from the above analysis, we first extracted the EC values within these regions and then correlated the EC values of each surviving region with the FMA‐HW scores in all stroke, PPH, and CPH patients using Spearman's correlation analysis. The cluster size for each subregion was estimated by Monte Carlo simulation^32^ using the RESTplus AlphaSim utilities (http://restfmri.net/forum/restplus). Finally, we conducted a pilot ROC curve analysis for each EC pattern displaying significant subgroup differences to assess whether the EC patterns might serve as objective neuroimaging metrics for the classification of patients with PPH and CPH.

## 3 RESULTS

### 3.1 Clinical and neuroimaging characteristics of the participants

In total, 680 patients experiencing stroke and 48 healthy controls were recruited during the study period. Due to the refusal of participants, 3 stroke patients and 1 healthy control had missing MRI data. Thus, we finally included 65 patients with left subcortical chronic stroke and 40 healthy controls for statistical analyses. Of the 65 stroke patients, 32 were classified as having PPH, and 33 were classified as having CPH. Table 1 shows the baseline characteristics of all participants. All of the stroke patients were right‐handed (54 male; 30 infarctions), with a mean age of 55.89 years old, disease course of 14.70 months, and hand deficits of 6.17 points. The distribution and proportion of age, stroke type, duration of illness and framewise displacement were comparable between the groups. However, a significant difference in the gender ratio (*p* = 0.009) was detected between the stroke patients and healthy controls. Moreover, the PPH patients had significantly higher FMA‐HW scores than the CPH patients (*p* < 0.001), while the lesion volumes showed the reverse relationship (*p* = 0.006).

**TABLE 1:** Clinical and neuroimaging data of participants recruited in this study

Baseline Characteristics	Whole Group Comparison		*p* Value	Subgroup Comparison		*p* Value
	Stroke (*n* = 65)	Controls (*n* = 40)		PPH (*n* = 32)	CPH (*n* = 33)	
Age (years)^a^	55.89 ± 9.71	55.12 ± 7.57	0.671	56.19 ± 10.53	55.60 ± 9.00	0.811
Gender (male:female)^b^	54:11	24:16	0.009	29:3	25:8	0.110
Hand dominance	Right	Right	‐	Right	Right	‐
Stroke type (ischemic: hemorrhagic)^b^	30:35	‐	‐	16:16	14:19	0.540
Duration of illness (months)	14.70 ± 16.07	‐	‐	15.31 ± 14.87	14.12 ± 17.36	0.305
Lesion hemisphere (left:right)	Left	‐	‐	Left	Left	‐
Lesion location	Subcortical	‐	‐	Subcortical	Subcortical	‐
Lesion volume (ml)	12.78 ± 9.50	‐	‐	9.45 ± 5.57	16.00 ± 11.33	0.006
FMA‐HW score	6.17 ± 6.67	‐	‐	11.25 ± 6.15	1.24 ± 1.22	<10^−9^
Framewise displacement (mm)	0.14 ± 0.08	0.11 ± 0.05	0.089	0.13 ± 0.07	0.14 ± 0.10	0.823

*Note*: Values are presented as the means ± standard deviations; the superscript *a* indicates the two independent samples *t* test, *b* indicates the chi‐square test, and all others are the Mann–Whitney test. PPH, partially paretic hand; CPH, completely paretic hand; FMA‐HW, Hand and Wrist subscale of the Fugl‐Meyer Assessment.

### 3.2 Disrupted EC patterns and their correlations with hand performance in all stroke patients

**FIGURE 4:**
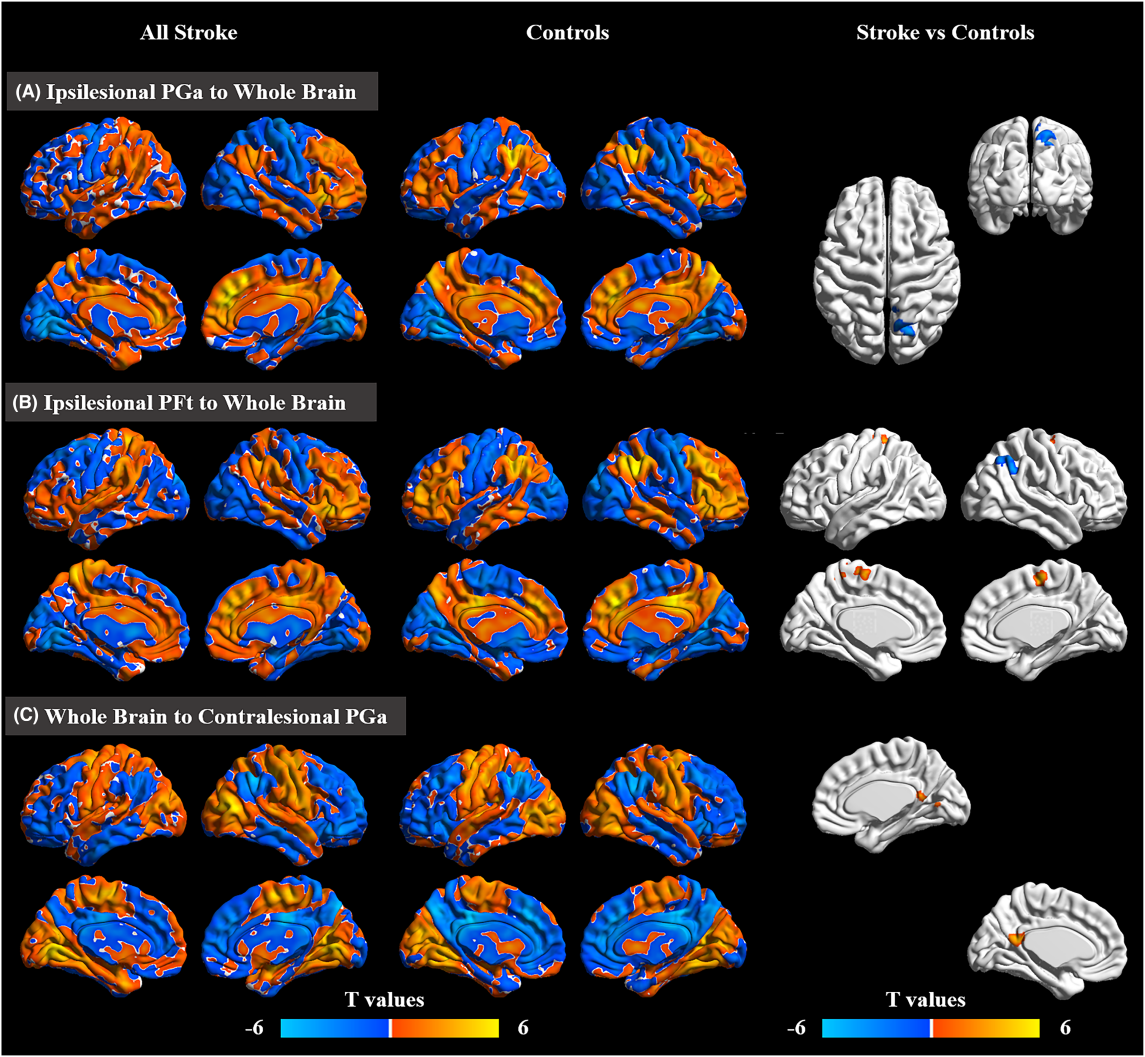
Disrupted effective connectivity in stroke patients compared with healthy controls. (A,B) Group differences in effective connectivity from seeds (ipsilesional PGa and ipsilesional PFt) to the whole brain. (C) Group differences in effective connectivity from the whole brain to the seed (contralesional PGa). AlphaSim corrected: *p* < 0.001

Compared with healthy controls, stroke patients showed increased EC from the ipsilesional PFt to the SMA and from the posterior cingulate gyrus to the contralesional PGa. However, compared with healthy controls, stroke patients showed decreased EC from the ipsilesional PGa to the contralesional superior parietal lobule and from the ipsilesional PFt to the contralesional IPL (Table 2, Figure 4).

**TABLE 2:** Significant EC differences in the IPL subregions between stroke patients and healthy controls and between stroke subgroups with different hand dysfunction

Group Differences in Effective Connectivity	MNI			Cluster	T Value
	X	Y	Z		
Stroke > Control					
Ipsilesional PFt to supplementary motor area	6	−15	60	120	4.46
Posterior cingulate gyrus to contralesional PGa	−6	−48	18	69	4.26
Stroke < Control					
Ipsilesional PGa to contralesional superior parietal lobule	21	−72	57	108	−4.35
Ipsilesional PFt to contralesional inferior parietal lobule	39	−66	57	103	−4.09
PPH < CPH					
Contralesional PFop to ipsilesional superior frontal gyrus	−15	57	27	65	−5.35

Abbreviations: CPH, completely paretic hand; EC, effective connectivity; IPL, inferior parietal lobule; PPH, partially paretic hand.

**FIGURE 5:**
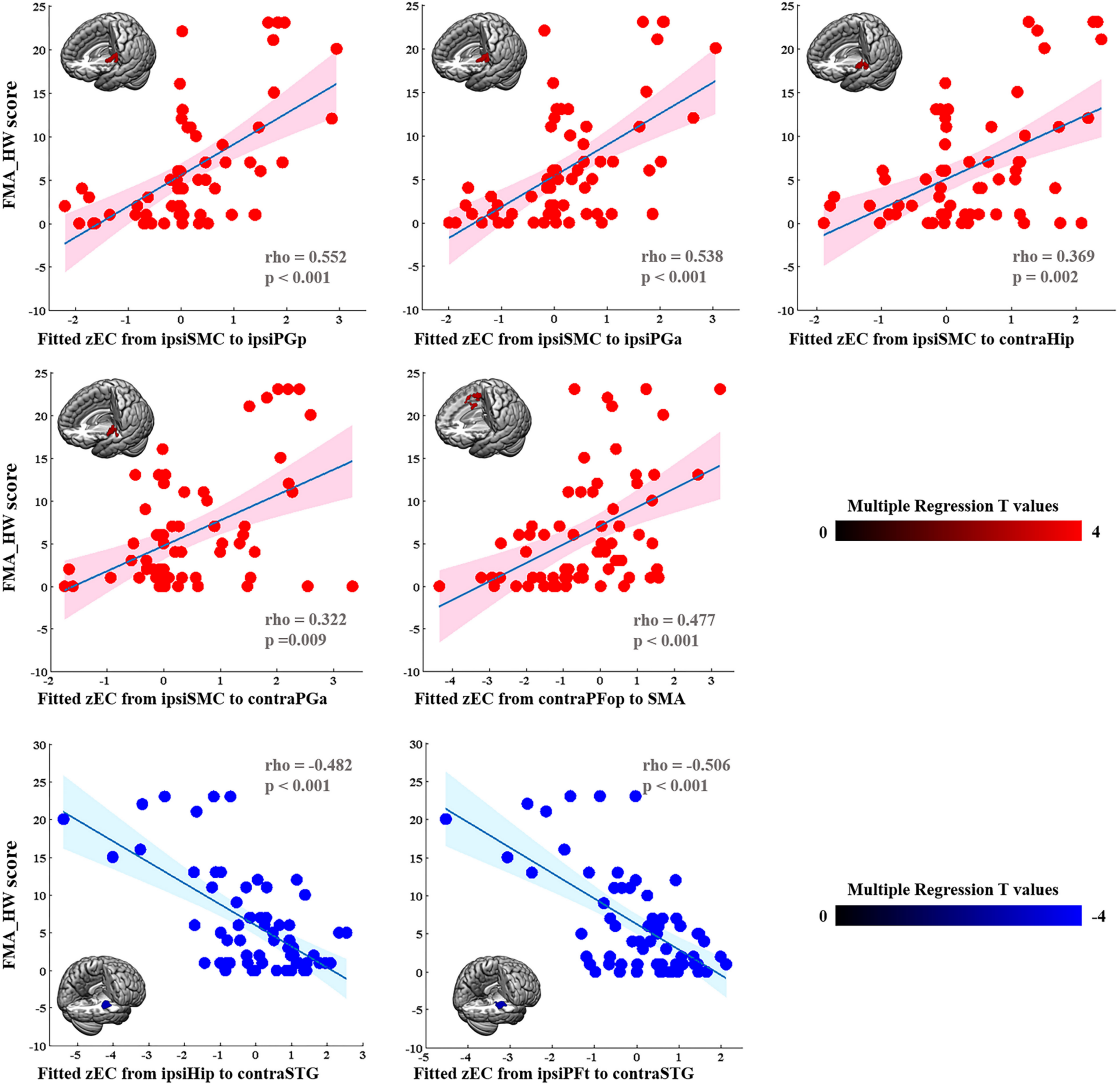
Correlations between connectivity patterns and paretic hand performance in all stroke patients. The correlations were conducted by multiple regression (AlphaSim corrected: *p* < 0.001) and Spearman's correlation analyses. contra, contralesional; FMA‐HW, Hand and Wrist Subscale of the Fugl‐Meyer Assessment; ipsi, ipsilesional; SMA, supplementary motor area; SMC, sensorimotor cortex; STG, superior temporal gyrus

The FMA‐HW scores were positively correlated with the EC values from the ipsilesional sensorimotor cortex (SMC) to the ipsilesional PGp (rho = 0.552, *p* < 0.001), ipsilesional PGa (rho = 0.538, *p* < 0.001), contralesional Hip (rho = 0.369, *p* = 0.002) and contralesional PGa (rho = 0.322, *p* = 0.009) and from the contralesional PFop to the SMA (rho = 0.477, *p* < 0.001). However, the FMA‐HW scores were negatively correlated with the EC values from the ipsilesional Hip (rho = −0.482, *p* < 0.001) and PFt (rho = −0.506, *p* < 0.001) to the contralesional superior temporal gyrus (STG) (Figure 5).

### 3.3 Disrupted EC patterns and their correlations with different hand dysfunction in stroke subgroups

**FIGURE 6:**
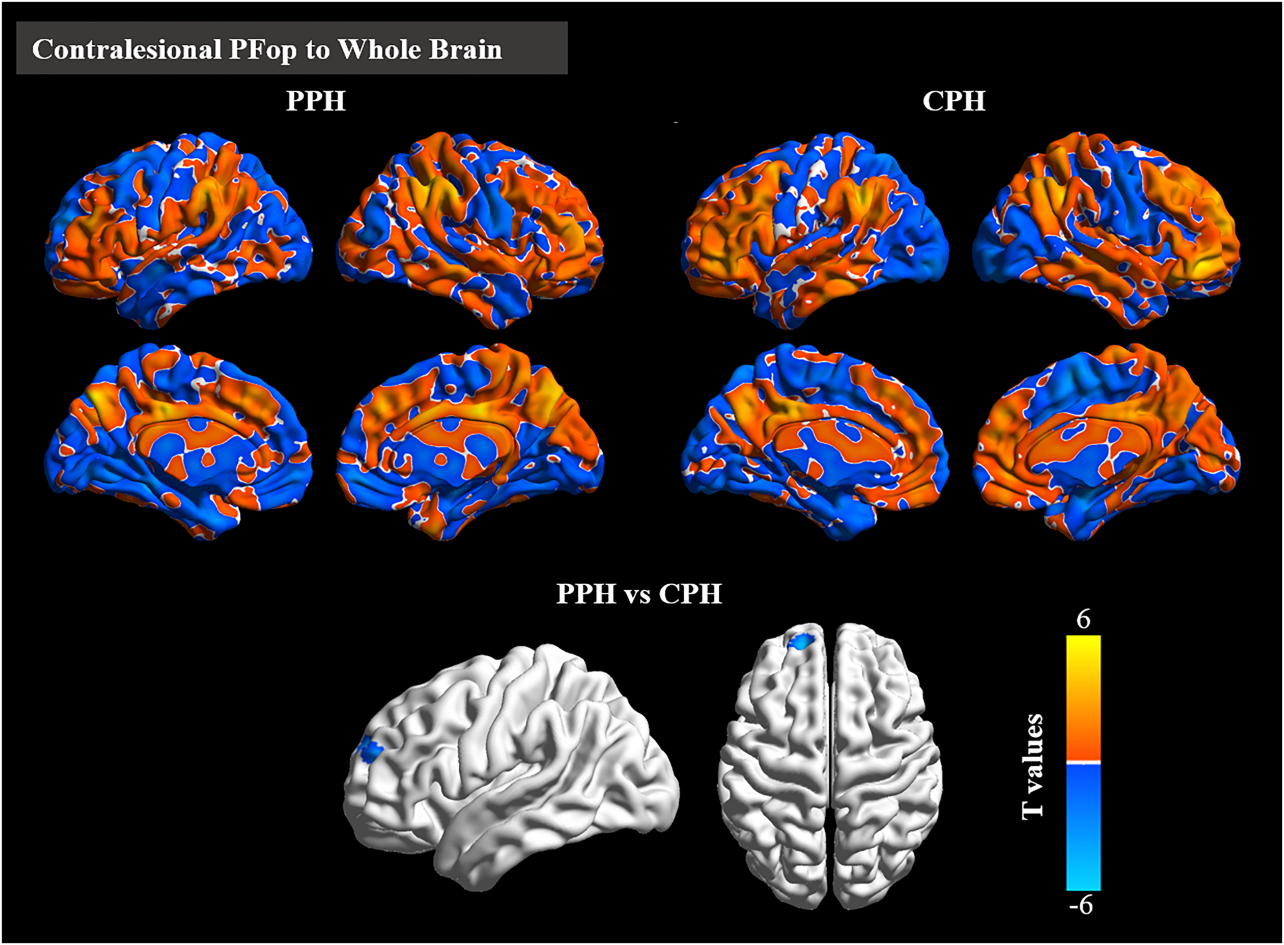
Disrupted effective connectivity between stroke patients presenting with PPH and CPH. Group differences in effective connectivity from the seed (contralesional PFop) to the whole brain. AlphaSim corrected: *p* < 0.001. CPH, completely paretic hand; PPH, partially paretic hand

Compared with CPH patients, PPH patients showed decreased EC from the contralesional PFop to the ipsilesional superior frontal gyrus (SFG) (Table 2, Figure 6).

**FIGURE 7:**
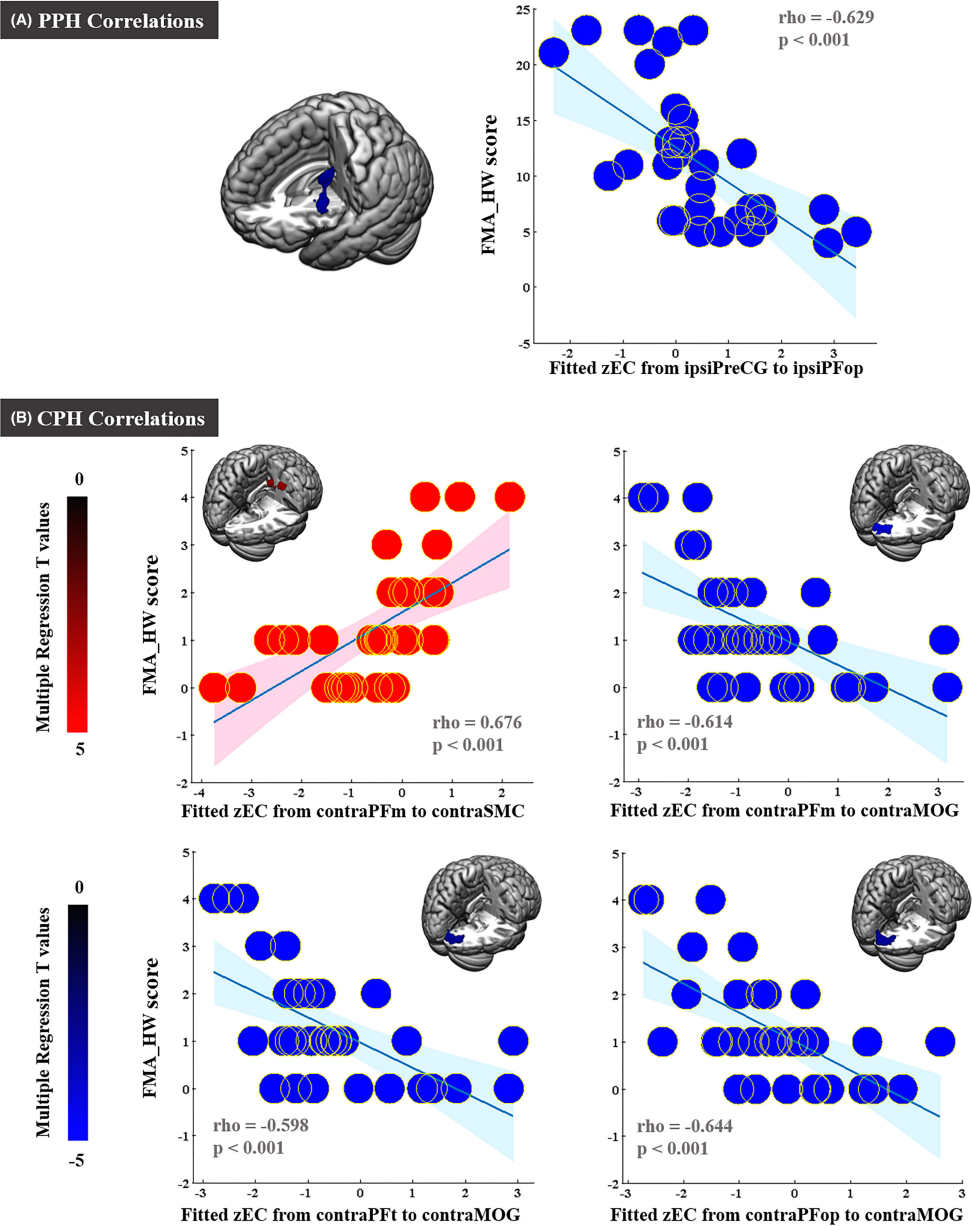
Correlations between connectivity patterns and different hand performance in stroke subgroups. The correlations were conducted by multiple regression (AlphaSim corrected: *p* < 0.001) and Spearman's correlation analyses. contra, contralesional; CPH, completely paretic hand; FMA‐HW, Hand and Wrist Subscale of the Fugl‐Meyer Assessment; ipsi, ipsilesional; MOG, middle occipital gyrus; PPH, partially paretic hand; PreCG, precentral gyrus; SMC, sensorimotor cortex

In patients with PPH, the FMA‐HW scores were negatively correlated with the EC values from the ipsilesional precentral gyrus (PreCG) to the ipsilesional PFop (rho = −0.629, *p* < 0.001). In patients with CPH, the FMA‐HW scores were positively correlated with the EC values from the contralesional PFm (rho = 0.676, *p* < 0.001) to the contralesional SMC. However, the FMA‐HW scores were negatively correlated with the EC values from the contralesional PFm (rho = −0.614, *p* < 0.001), PFt (rho = −0.598, *p* < 0.001) and PFop (rho = −0.644, *p* < 0.001) to the contralesional middle occipital gyrus (MOG) (Figure 7).

### 3.4 Sensitivity and specificity of EC metrics for classifying different hand dysfunctions

**FIGURE 8:**
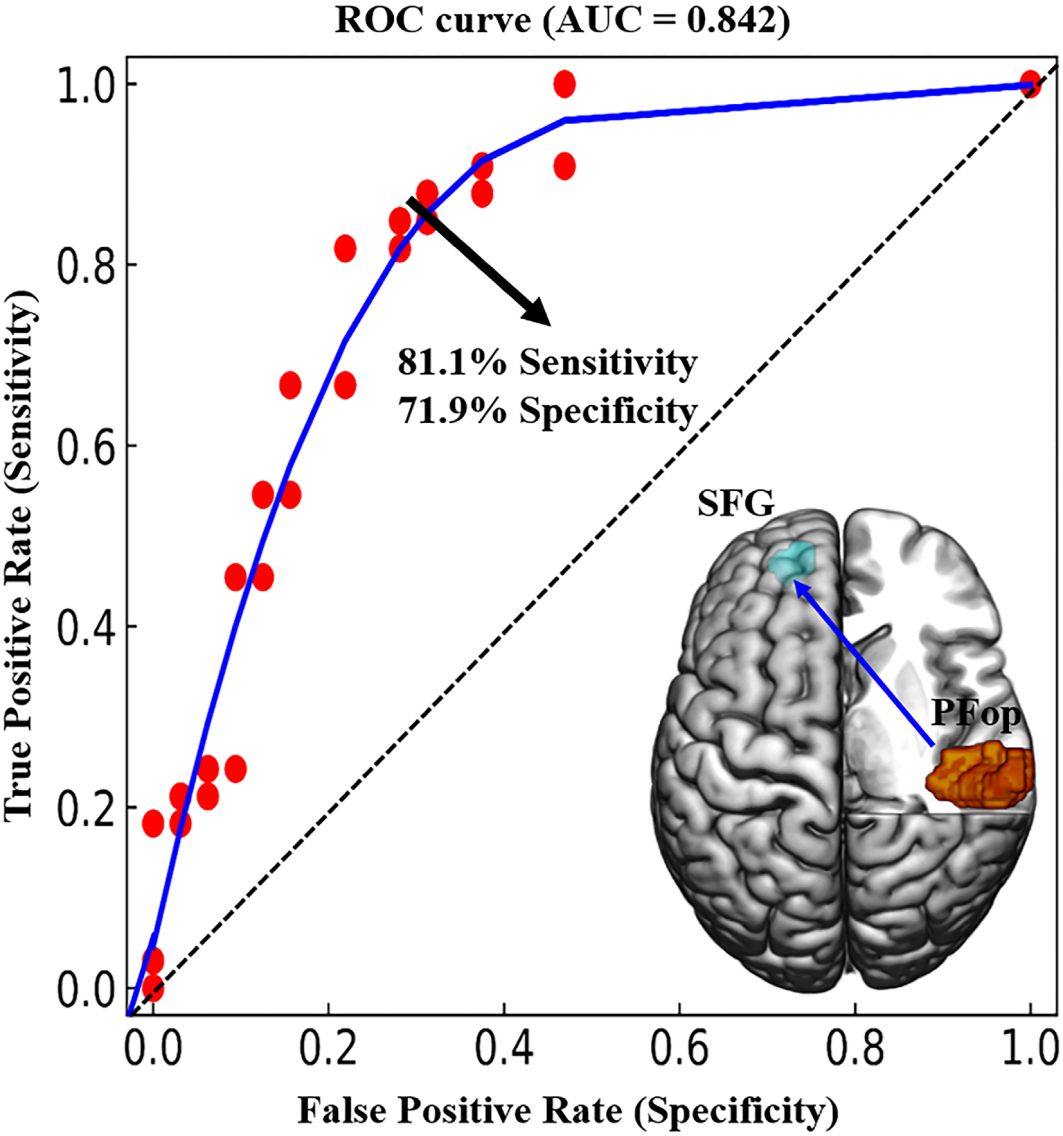
Classification of stroke patients who presented with different hand dysfunctions. The mean effective connectivity values from the contralesional PFop to the ipsilesional SFG distinguished patients with a partially paretic hand from patients with a completely paretic hand with high sensitivity and specificity. AUC, area under the curve; ROC, receiver operating characteristic; SFG, superior frontal gyrus

The mean EC strength from the contralesional PFop to the ipsilesional SFG showed the greatest accuracy (area under the curve = 0.842, *p* < 0.001) for distinguishing patients with PPH from patients with CPH. Specifically, using a cutoff value of 0.006, 25 of the 32 patients with PPH and 26 of the 33 patients with CPH were classified exactly, producing a sensitivity of 81.1% and a specificity of 71.9% (Figure 8).

## 4 DISCUSSION

Targeting of individualized brain regions is critical for the efficacy of noninvasive neuromodulation in rehabilitation practice.^33^, ^34^, ^35^ However, knowledge about the potential neuroimaging targets of hand dysfunction after chronic stroke has been limited. Here, we report for the first time selectively disrupted EC patterns of IPL subsystems in patients experiencing chronic stroke and correlated these specific EC patterns with paretic hand performance. Moreover, we identified that EC strength from the contralesional PFop to the ipsilesional SFG distinguished patients with different types of hand dysfunction. These findings indicated that specific reorganizations of IPL subsystems are disrupted and associated with hand dysfunction after chronic stroke.

### 4.1 Disrupted EC patterns following chronic stroke and their associations with hand performance

Cytoarchitectonic studies have divided the human IPL into the supramarginal gyrus (including the subregions of PF, PFcm, PFm, PFt, PFop) and angular gyrus (including the subregions of PGp, PGa).^18^ Specifically, the anterior IPL (PFt and PFop) is linked with the sensorimotor and salience networks, the central IPL (PFm) is linked with the executive control network, and the posterior IPL (PGp and PGa) is linked with the default mode network,^19^, ^21^ corresponding to the findings of receptor architectonics in IPL.^31^ In chronic stroke patients, functional connectivity between the ipsilesional primary motor cortex (M1) and the ipsilesional IPL (corresponding to middle IPL subregions) was positively correlated with hand performance.^36^ For stroke patients with M1 lesions, the improvement of hand dexterity was associated with compensatory connectivity among perilesional core motor regions.^37^ However, in this study, the EC values from the ipsilesional SMC to the ipsilesional PGp and PGa, as well as the contralesional Hip and PGa, were positively correlated with hand performance in chronic stroke patients. These findings suggest that causal influences from the ipsilesional SMC to posterior or rostral dorsal subsystems of the IPL could compensate for M1 dysfunction and support voluntary movements of the paretic hand in patients after chronic stroke.

The extended mirror neuron network includes the ventrolateral prefrontal cortex, dorsal/ventral premotor cortex, M1, pre‐SMA, anterior cingulate cortex and rostral IPL, which become active during both the execution and observation of actions.^38^ The SMA, with mirror‐like properties, contributes to the preparation and execution of hand movements.^39^ When the corticospinal tract is damaged, the SMA might support or take over M1 functionality during reaching and grasping after stroke.^40^ In well‐recovered patients following stroke, connectivity from the contralesional prefrontal cortex to the SMA correlates with motor performance during motor imagery.^41^ When inhibiting the contralesional M1 by transcranial magnetic stimulation, Grefkes et al. found that increased couplings from the ipsilesional SMA to M1 were associated with motor recovery.^22^ As a classical and crucial mirror region, the anterior IPL plays important roles in sending visuomotor information to the sensorimotor system to guide action execution.^42^ Interestingly, in this study, we observed increased EC from the ipsilesional PFt to the SMA in chronic stroke patients compared with healthy controls. Furthermore, paretic hand performance was positively correlated with the EC values from the contralesional PFop to the SMA. These findings indicate that increased causal influences within the parietal‐motor mirror circuits could contribute to the motor output of the paretic hand in patients after chronic stroke.

The IPL and its projections to the superior temporal sulcus constitute a core mirror network for imitation, in which the superior temporal sulcus provides a higher‐order visual description of the imitated action, whereas the parietal component relates to the motor aspects of the imitated action.^43^ As shown in our previous study, the EC values from the inferior temporal gyrus to primary somatosensory cortex_1 within the ipsilesional hemisphere are negatively correlated with hand performance after chronic stroke.^14^ Similar to these findings, we also found that the EC values from the ipsilesional Hip and PFt to the contralesional STG showed negative correlations with hand performance in chronic stroke patients. These findings suggest that recruitment of the parietal–temporal subsystems might represent maladaptive reorganization and could be associated with poor hand dysfunction after chronic stroke.

### 4.2 Distinct EC patterns across stroke subgroups and their associations with hand performance

The mirror neuron network is a particular higher‐order motor system, and the key frontal and parietal components form neural circuits devoted to the visuomotor transformation necessary for reaching and grasping during hand movements.^44^ The SMC includes both the PreCG and postcentral gyrus, and M1 within the PreCG is a key hotspot driving the voluntary movements of the contralateral hand.^45^ Zhao et al. reported that the contralesional PreCG displayed significant interactions between the frequency band and the severity of hand dysfunction in chronic stroke patients.^46^ Using intermittent theta burst stimulation to modulate the ipsilesional M1, Volz et al. observed a significant recovery of grip strength in patients with subacute stroke.^47^ In this study, we revealed that the EC values from the ipsilesional PreCG to the ipsilesional PFop were negatively correlated with hand performance in PPH patients. Our previous study reported that CPH patients exhibited increased intranetwork connectivity in the contralesional SMC, and the increased connectivity was negatively associated with hand performance.^13^ Here, we found that the EC values from the contralesional PFm to the contralesional SMC were positively correlated with hand performance in CPH patients. These findings indicate that functional reorganizations between the specific IPL subregions and sensorimotor systems within the ipsilesional hemisphere play negative roles in patients with PPH, while they play positive roles within the contralesional hemisphere in patients with CPH. This dissociation phenomenon could be related to severe corticospinal tract damage in patients with CPH because a larger lesion load is often accompanied by increased activation and reorganization within the contralesional sensorimotor system.^48^, ^49^, ^50^

Emerging evidence has indicated that the occipitotemporal cortex should be considered a potential component of the human mirror neuron network, which is involved in understanding the actions of others.^51^ The MOG processes several important types of visuomotor information, including shape detection, space depth and movement distance.^52^ Our previous study reported that the EC values from the ipsilesional primary motor cortex_4p to the contralesional calcarine were negatively correlated with hand performance in chronic stroke patients presenting with severe hand deficits.^14^ Additionally, one longitudinal rs‐fMRI study also revealed decreased functional connectivity between the contralesional M1 and occipital cortex in stroke patients during spontaneous recovery.^53^ In this study, we found that the EC values from the contralesional PFm, PFt and PFop to the contralesional MOG were negatively correlated with hand performance in patients with CPH. These findings suggest that abnormal causal influences from the anterior/middle IPL subregions to MOG within the contralesional hemisphere could indicate poor hand dysfunction in patients after chronic stroke.

### 4.3 Classifying stroke patients presented different hand dysfunction

Based on rs‐fMRI and machine learning approaches, Rehme et al. classified stroke patients and healthy controls with 82.6%–87.6% accuracy.^54^ However, classifying stroke patients with different severities of hand dysfunction remains unsettled. Here, we used the mean EC strength from the contralesional PFop to the ipsilesional SFG to distinguish PPH patients from CPH patients with accuracy of 84.2%. M1 activation,^55^ motor pathway integrity^56^ and serum biochemistry (e.g., direct bilirubin)^57^ have been used as biomarkers to predict long‐term outcomes following stroke. Using 1‐Hz repetitive transcranial magnetic stimulation to inhibit the contralesional M1, Nowak et al. reported that baseline overactivation of the contralesional dorsal premotor cortex predicted improvement in the paretic hand.^58^ From the connectivity perspective, Grefkes et al. demonstrated that improved hand performance was significantly associated with decreased interhemispheric inhibition originating from the contralesional M1 during affected hand movement.^22^ These findings imply that neuroimaging biomarkers combined with noninvasive brain stimulation might help in the development of personalized neurorehabilitation in chronic stroke patients with hand dysfunction.

### 4.4 Limitations and future perspectives

First, the baseline gender distribution was unbalanced between the stroke patients and healthy subjects, which could be attributed to the higher incidence of stroke in men than in women.^59^ However, men might have better performance than women on motor recovery after rehabilitation.^60^ To avoid this source of bias, gender was regressed out in all statistical analyses. Second, although most spontaneous recovery tends to occur within the first 3 months following stroke onset,^61^ given the heterogeneity in disease course and lesion size for stroke patients, we also regressed out these parameters in subgroup analyses. Third, both ischemic stroke and hemorrhagic stroke were integrated, but this study is still more homogeneous than previous studies that included ischemic and hemorrhagic, left and right and cortical and subcortical stroke. More importantly, although ischemic and hemorrhagic stroke have different pathophysiological mechanisms during the acute and subacute stages, motor deficits are primarily determined by the structural damage to motor pathways but not the stroke type during the chronic stage.^62^, ^63^ Fourth, the use of recently proposed imaging methods to evaluate cerebral edema formation^64^ and microvasculature morphology^65^ could help to supplement the neural mechanisms of hand dysfunction following acute to subacute stroke. Finally, imaging studies involving thrombectomy,^66^ preclinical components (e.g., glyceryl trinitrate)^67^ and thrombolytic drugs^68^ after acute stroke have been limited; thus, it would be interesting to explore this new field in the future.

## 5 CONCLUSION

In summary, we reported specifically disrupted IPL subsystems in patients with chronic stroke presenting hand dysfunction. Moreover, we clarified the brain‐behavior associations between the specific EC patterns and paretic hand performance in all patients experiencing chronic stroke and across stroke subgroups. Finally, we found that the mean EC strength from the contralesional PFop to the ipsilesional SFG could well classify stroke patients diagnosed with PPH and CPH. These findings could be beneficial for clarifying the neural basis of hand dysfunction after chronic stroke.

## AUTHOR CONTRIBUTIONS

CZT involved in development and design of the study concept; FWL, CCC, and ZFB involved in data acquisition and analysis; FWL and CZT involved in initial manuscript writing. All of the authors revised and confirmed the final version of this article

## CONFLICT OF INTEREST

The authors declare no disclosures related to this work.

## Supporting information


Figure S1
Table S1

## Data Availability

Data are available upon request from the corresponding authors.
