# Supplementary material for: Specific subsystems of the inferior parietal lobule are associated with hand dysfunction following stroke: A cross‐sectional resting‐state fMRI study
Source: CNS Neurosci Ther. 2022 Aug 23;28(12):2116–28. doi: 10.1111/cns.13946 (PMC9627383; doi:10.1111/cns.13946)
Supplement: Supplementary file 1 — Figure S1 Table S1 [file CNS-28-2116-s001.docx]

**Fig. A. 1** The lesion location of each PPH and CPH patient is shown in T2-weighted axial images. Left indicates the ipsilesional hemisphere.

**
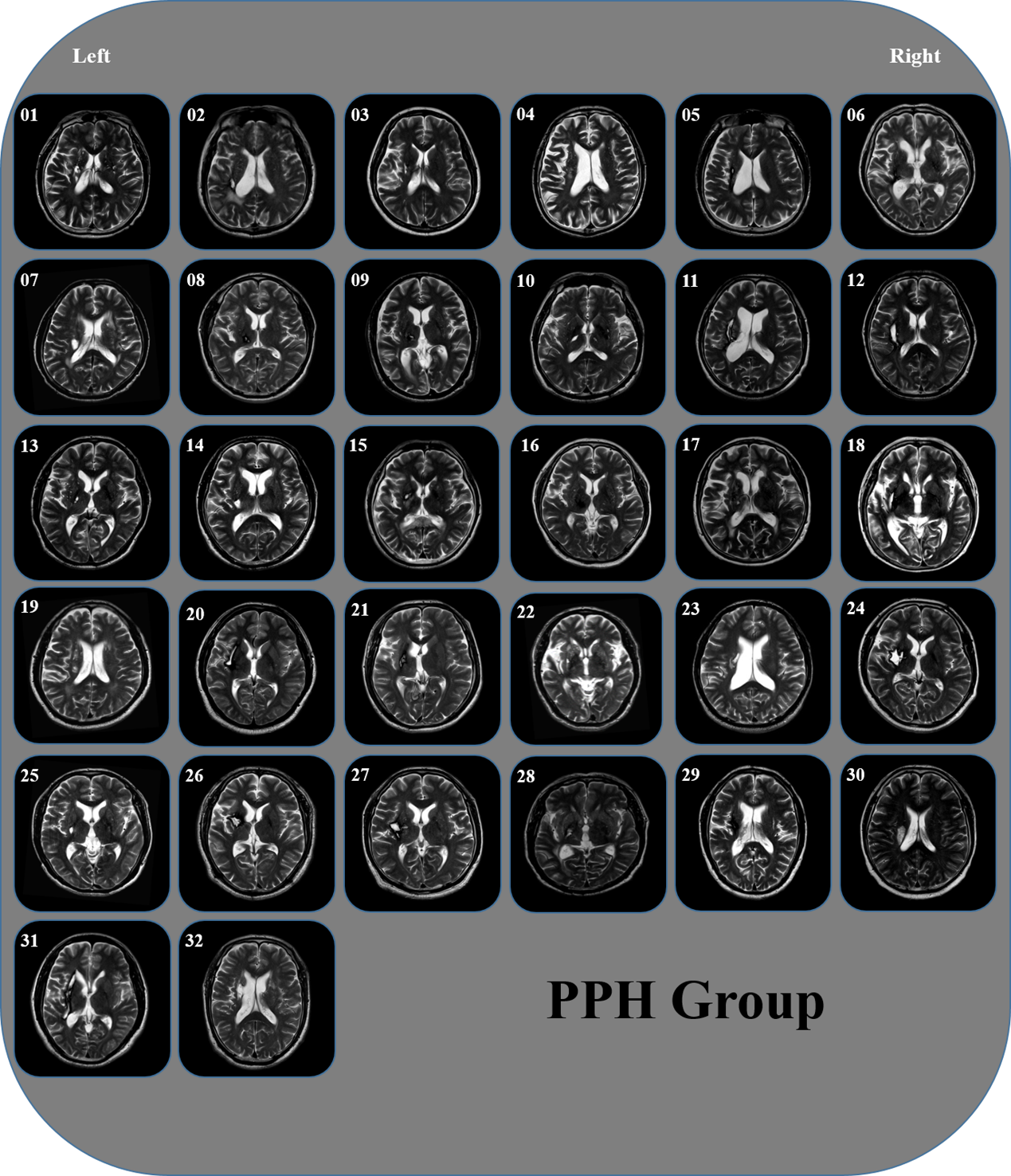
**

**
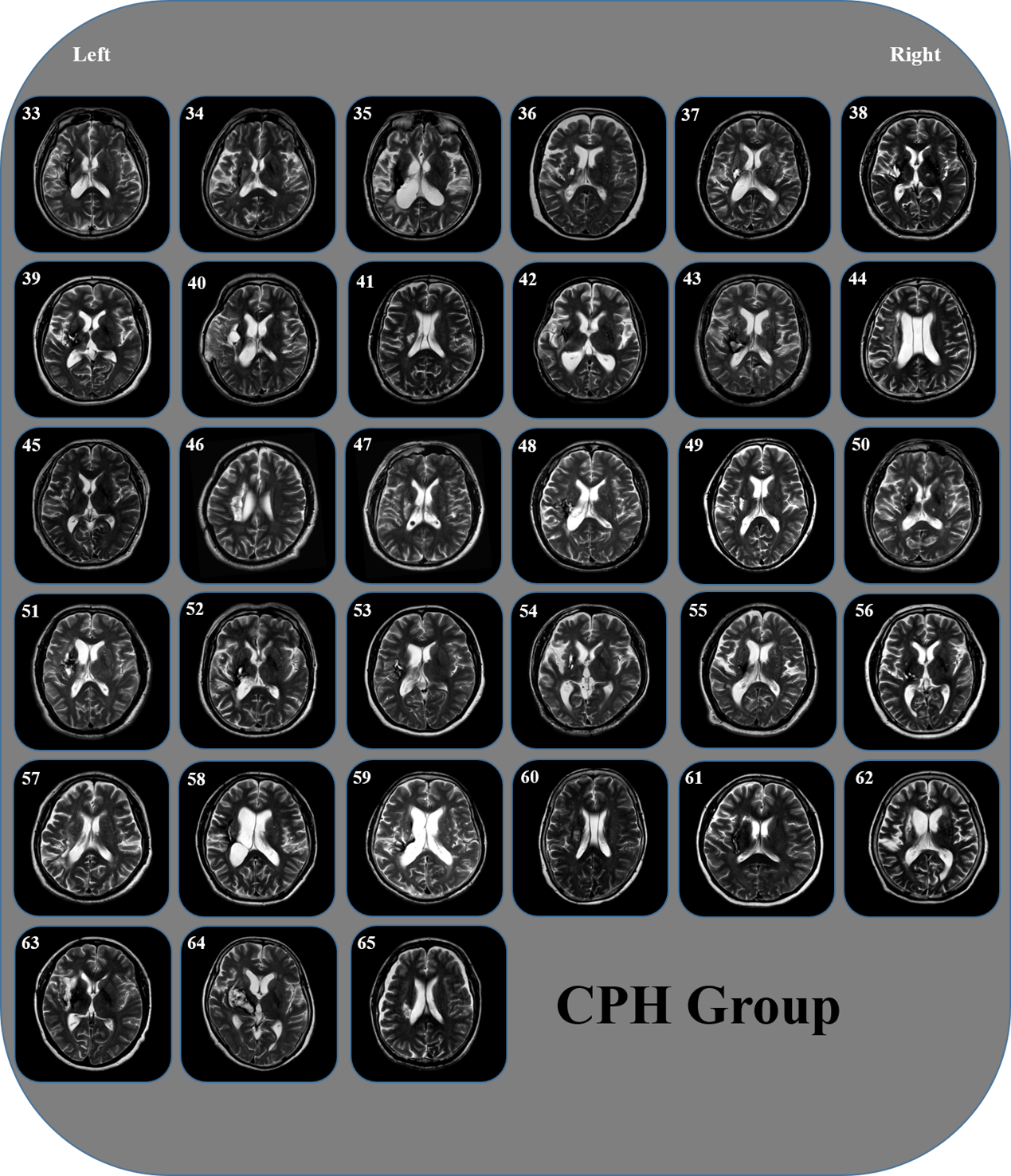
**

**Table A.1** Paretic Hand Scale.

| **Action items** | **Illustration** | **Hand function classification** | **Evaluation criteria** |
| --- | --- | --- | --- |
| 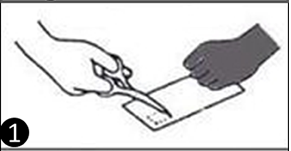 | The affected hand stabilizes a piece of paper on the table, and the unaffected hand uses a shear to cut the paper. | Disabled hand | Could not complete any activities. |
| 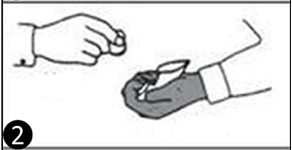 | The affected hand holds a wallet, and the unaffected hand takes a coin from the wallet. | Assistant hand C | Finished one of the five activities. |
| 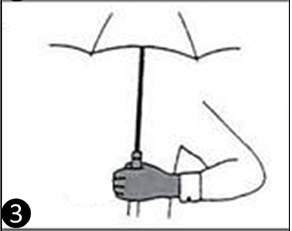 | The affected hand holds an unfolded umbrella in the air for at least 10 seconds. | Assistant hand B | Finished two of the five activities. |
|  |  | Assistant hand A | Finished three of the five activities. |
| 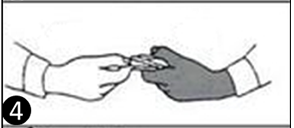 | The affected hand controls a nail scissor to trim nails of the unaffected hand. | Practical hand B | Finished four of the five activities. |
| 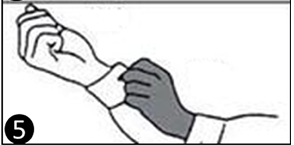 | The affected hand buttons the cuff of the unaffected side. | Practical hand A | Completed all of the five activities. |

**Note.** The hand in dark denotes the affected hand.
